# BRIC Health Systems and Big Pharma: A Challenge for Health Policy and Management

**DOI:** 10.15171/ijhpm.2017.145

**Published:** 2018-01-02

**Authors:** Victor G. Rodwin, Guilhem Fabre, Rafael F. Ayoub

**Affiliations:** ^1^Robert F. Wagner Graduate School of Public Service, New York University, New York City, NY, USA.; ^2^Université Paul Valéry Montpellier 3, IRIEC EA 740, Montpellier, France.; ^3^Wagner School of Public Service, New York University, New York City, NY, USA.

**Keywords:** Pharma, BRIC, Health Systems, Pharmerging, Healthcare, Population Health, Brazil, Russia, India, China

## Abstract

BRIC nations – Brazil, Russia, India, and China – represent 40% of the world’s population, including a growing aging population and middle class with an increasing prevalence of chronic disease. Their healthcare systems increasingly rely on prescription drugs, but they differ from most other healthcare systems because healthcare expenditures in BRIC nations have exhibited the highest revenue growth rates for pharmaceutical multinational corporations (MNCs), Big Pharma. The response of BRIC nations to Big Pharma presents contrasting cases of how governments manage the tensions posed by rising public expectations and limited resources to satisfy them. Understanding these tensions represents an emerging area of research and an important challenge for all those who work in the field of health policy and management (HPAM).

BRIC nations – Brazil, Russia, India, and China – represent 40% of the world’s population, including a growing aging population and middle class with an increasing prevalence of chronic disease.^1^ As in healthcare systems among G7 nations, they rely increasingly on prescription drugs, but they differ from these mature nations with respect to healthcare expenditure growth. Although BRIC nations spend far less than the average of G7 nations on healthcare, as a percent of gross domestic product (GDP), and accounted for a much smaller share of the global market in 1995 (9% in $PPP [purchasing power parity]), since then this share has almost doubled (16% in 2012).^2^ BRIC nations’ healthcare expenditure growth rates, between 1995-2013, exceeded those of G7 nations whose percentage share of global health spending fell, mostly due to the growth of healthcare expenditure in BRIC nations.^2^ Likewise, pharmaceutical multinational corporations (MNCs), henceforth Big Pharma, have seen their highest revenue growth in BRIC nations whose markets they refer to as “pharmerging.”^3^ Since 2012, IMS Health estimates Big Pharma’s compound average annual revenue growth rates at 13% in comparison to 2% in the top eight mature markets.^3^ During this same period, IMS estimates a 17% compound annual growth rate, in China, and an average of 12% for Brazil, Russia, and India.^3^

Big Pharma has always sought to maximize its sales of branded drugs and protect its intellectual property rights. Since the AIDS crisis, however, Brazil and India turned to compulsory licensing to make life saving drugs available to their populations. Also, Big Pharma had to contend with patent expiration for half of the world’s 100 best selling medicines.^4^ In addition, in mature markets, the growth of less expensive generic drugs and the strengthening of regulations governing the efficacy and safety of new medicines led Big Pharma to introduce new strategies to maintain its profit margins and global revenue growth rates.^5^ The so-called patent cliff exacerbated Big Pharma’s traditional business model of spending more on marketing and promotion of “me-too medicines” than on research and development.^4,6-9^ In the context of higher sales revenue growth in BRIC nations, Big Pharma has adapted their business models to these pharmerging markets.

BRIC nations present a number of risks to Big Pharma. They all have less well developed healthcare systems, a shortage of expertise in some domains, a history of problems with quality control, economic crisis, and corruption.^5^ On the other hand, they may make it possible to lower the costs of clinical trials and other aspects of drug development. Moreover, periods of prosperity in BRIC nations, and aging middle-class populations, have led to rapidly changing disease patterns.^1^ Of course, there are striking differences among BRIC nations with respect to population health,^1^ the financing and organization of health services, and healthcare coverage.^10^ Their response to Big Pharma presents contrasting examples of the tensions posed by rising public expectations and limited resources to satisfy them. Understanding these tensions represents an emerging area of important research and a challenge for all those who work in the field of health policy and management (HPAM).

## Population Health and Healthcare Systems

Beyond the problems of HIV/AIDS and tuberculosis (TB), which challenged Big Pharma’s intellectual property regime, BRIC nations now confront the rise of non-communicable disease (NCD) associated with rising urbanization and pollution, especially in China and India (Table 1). Russia lags behind Brazil and China with respect to life expectancy at birth and mortality from NCD (Table 1). India still lags far behind Brazil, China, and Russia with respect to life expectancy at birth, which reflects its significantly higher mortality from communicable disease. As for NCD, India’s death rates are higher than Brazil and China, but lower than Russia.

**Table 1:** Population Health and NCD: Indicators in BRIC Nations, 2012-2017

**Indicator**	**Brazil**	**Russian Federation**	**India**	**China**
Population (millions)^a^	205.9	143.8	1309	1397
Life expectancy at birth^b^	75.2	70.9	68.3	76.1
Age standardized mortality rate from NCD per 100 000^c^	450	716	600	550
Cancer mortality per 100 000 population^d^	103.7	122.6	64.5	122.2
Probability of dying (ages of 30-70) from CVD, cancer, diabetes, chronic respiratory disease (%)^c^	17	29	23	18

Abbreviations: NCD, non-communicable disease; CVD, Cardiovascular disease; BRIC, Brazil, Russia, India, and China. Sources: ^a^ United Nations Population Division (2017). World Population Prospects. ^b^ World Bank (2015). ^c^ WHO. Global Health Observatory Data Repository (2015). ^d^ Cancer Research UK – Worldwide Cancer Mortality Statistics (2012).

BRIC nations’ contrasting models of healthcare financing and organization reflect political and institutional features that are important to understanding their response to Big Pharma. Russia, although the wealthiest, among BRIC nations, measured by GDP per capita, in PPP prices, spends significantly less on healthcare (6.1% of GDP) than Brazil (8.3%) (Table 2). China, third in GDP per capita, spends only 5.6% of its GDP on healthcare, while India spends only 4.8%. Most striking is the relative share of public and private expenditure on healthcare. India’s public share of healthcare expenditure is only 29% (1.4% of GDP) in contrast to 55% in China (3.1% of GDP), 60% in Russia (3.7% of GDP) and 46% in Brazil (3.8% of GDP). Likewise, India is the outlier with respect to out-of-pocket (OOP) payment by patients, as a share of total healthcare expenditures: 65.6% in India in contrast to 25.5% in Brazil, 45.8% in Russia and 34.6% in China (Table 2).^11^

**Table 2:** Healthcare Expenditure in BRIC Nations, 2014-2016

	**Brazil**	**Russian Federation**	**India**	**China**
GDP per capita in PPP^a^	16096	24805	5855	12880
GNI per capita^b^	14810	22540	6490	15500
Human development index^c^	0.754	0.804	0.624	0.738
Gini index^d^	51.3	37.7	35.2	42.2
Public expenditure on health (% of GDP)^e^	3.8	3.7	1.4	3.1
Private expenditure on health (% of GDP)^e^	4.5	2.4	3.4	2.5
Total expenditure on health (% of GDP)^e^	8.3	6.1	4.8	5.6
OOP health expenditure (% of private health expenditure)^e^	47.2	88	95.9	72.3
OOP health expenditure (% of total healthcare expenditure)^e^	25.5	45.8	65.6	34.6
OOP health expenditure per capita in PPP^f^	$343	$506	$90	$165

Abbreviations: OOP, Out-of-pocket; PPP, purchasing power parity; GDP, gross domestic product; BRIC, Brazil, Russia, India, and China; GNI, Gross national income. ^a^ World Economic Outlook Database, International Monetary Fund (2014). ^b^ The World Bank (2016). ^c^ HDI is a composite statistic (composite index) of life expectancy, education, and per capita income indicators, Human Development Report UNDP. (2015) ^d^ GINI index of 0 represents perfect equality while an index of 100 implies perfect inequality. World Bank. (Brazil and Russia-2015 China-2012 India-2011) ^e^ The World Bank (2014). ^f^ Reference 11.

## Market Access for Big Pharma

Big Pharma’s largest and fastest growing market is obviously in China due to its size and economic growth.^12^ China’s annual growth in per capita healthcare expenditure over the period 1995-2012 was 10.4% in comparison to Brazil’s (3.3%), India’s (6.4%) and Russia’s (5.4%).^11^ Although Brazil, India, and Russia currently have similar sized pharmaceutical markets, India stands out as the nation with the lowest per capita pharmaceutical expenditures (Table 3).^13^ In terms of Big Pharma’s presence within each nation, and the extent to which the population has access to patented drugs, Brazil stands out as the nation with the highest share of its total expenditure on these medicines (47%), in contrast to China (22%), Russia (21%), and India (9.3%).^14^

**Table 3:** BRIC Pharmaceutical Market, 2016

	**Brazil**	**Russian Federation**	**India**	**China**
Pharmaceutical sales (in US$ Billion)^a^	18.38	15.4	17.45	116
Per capita (in US$)^a^	88	107	13	8.3
Pharmaceutical value imported (in US$ Billion)^b^	6.39	8.9	1.69	20.77
% Imported pharmaceutical sales^a^	34.76	57.79	9.68	17.9
% Expenditure on patented drugs^a^	47	21	9.3	22
Pharmaceutical spend as % of total health expenditure (2009)^c^	25.1	20.3	40.9	42.5

Abbreviation: BRIC, Brazil, Russia, India, and China. Sources: ^a^ International Trade Administration. 2016 Top Markets Report – Pharmaceuticals. ^b^ ITC. Calculations based on UN COMTRADE statistics. ^c^ Reference 13.

With respect to their capacity to assure domestic production of pharmaceuticals, both generic and branded generics, Russia is the most dependent on imported medicines, as a share of its total pharmaceutical sales (57.8%), in contrast to Brazil (34.7%), China (17.9%), and India (9.7%). These differences, among BRIC nations, reflect many factors, including the size of their markets for pharmaceuticals, their industrial policies with respect to domestic production of pharmaceuticals, the terms on which they have negotiated Big Pharma’s market access, and the extent to which they provide their populations with publicly financed healthcare and prescription drug coverage.^5,15,16^

## Brazil

Since 1988, when Brazil’s constitution established a right to healthcare, and two years later adopted the Unified Health System (*Sistema Unico de Saude* - SUS), the Ministry of Health led a campaign to provide universal health coverage (UHC), backed up by strong federal, state and local government support to implement the policy. Since 2002, consistent with this goal, Brazil’s Farmacia Popular provides prescription drug coverage, based on a national list of essential drugs, for all patients suffering from hypertension, diabetes, asthma and a number of other conditions.^17^ Medications have also included imports, and more recently, under the banner of judicialization, the poor, as well as middle class patients have taken legal measures to assure access to a high volume of medicines that few nations now supply to their population.^18^

Brazil has also been a leader in the use of compulsory licensing to promote access to essential medicines, especially with respect to HIV/AIDS. By volume, domestic companies hold around 70% of the pharmaceutical market.^19^ With respect to revenue, however, Big Pharma now controls about one-half of the market. This figure underestimates its presence as MNCs have been active in mergers and acquisitions of domestic companies.

In the present context of economic recession, the government is attempting to impose spending controls through centralization of purchases and strengthening regulation of pharmaceutical prices, eg, ANVISA’s (Agência Nacional de Vigilância Sanitária) denials to provide some high cost prescription drugs not included in its guidelines.^20^ This may well reduce the growth of publicly financed pharmaceutical expenditures, but Brazil’s population appears in a stronger position to demand these benefits than their counterparts in Russia, India, and China.

## Russia

As in Brazil, Russians have a constitutional right, in principle, to free healthcare and every citizen is assigned to a polyclinic based on their place of residence. In practice, however, outpatient prescription drugs are excluded from coverage for most of the population, which results in high OOP payments. The Vital Drug List includes essential drugs covering mostly seniors, the disabled, children and pregnant women. The largest share of these medicines is imported from abroad and are free, in principle, in hospitals. However, this sector represents only about 20 percent of all prescription drugs sold.^21^ The generic drug market accounted for over 80% of the pharmaceutical market in volume (and almost 50% in value) in 2006. The gap between the volume and value of this market indicates a pricing problem because relatively high prices are paid to manufacturers who are not known for developing new molecules.^21^

In the context of international sanctions, and strong reliance by Russia on pharmaceutical imports, the government announced an import substitution strategy to ensure that local drug production covers one-half of generic drugs by 2017, and half of all innovative drugs by 2020.^22^ Nonetheless, Russia’s pharmaceutical sector today is conspicuous for its continued reliance on imports and the relative underdevelopment of domestic production. Pharmaceuticals produced by Big Pharma rank at the top of all imports to Russia.^23^ Measured in US dollars, due to the sanctions and devaluation of the Rouble, domestic consumption of pharmaceuticals sold by Big Pharma has decreased. In 2015, in contrast to its BRIC counterparts, the volume of pharmaceutical “packets” on Russia’s retail market declined to their levels in 2006.^24^

## India

In contrast to the government’s weak involvement in India’s healthcare system, with regard to the pharmaceutical sector, the Indian government has promoted a strong manufacturing sector following the Indian Patent Act of 1970, which denied product patents and recognized only process patents. This made it possible for domestic manufacturers to develop a competitive pharmaceutical sector with far lower production costs than those of Big Pharma, and eventually to serve as the world’s factory for generic medicines. In terms of volume, India’s pharmaceutical sector for generic prescription drugs accounts for 20% of global export volume^25^ and 40% of all generic and over-the-counter pharmaceuticals sold in the United States.^26^

Big Pharma has challenged India’s Supreme Court and Intellectual Property Appellate Board for their failure to grant more patents and for issuing compulsory licenses.^27^ However, India continues to limit the influence of Big Pharma and instead promotes its own manufacturers of generic medicines. One might presume that such policies would reduce pharmaceutical prices and thereby improve access to drugs for Indian patients. However, despite the low production costs, domestic companies often sell their generics with sales margins often as high as 1000% to 4000% of their costs.^28,29^ Although the government had announced free access to medicines in public health facilities as a goal in 2012, this objective was abandoned in 2015.^30^

As one of the most privatized healthcare systems, worldwide, in 2013 the government authorized the National Pharmaceutical Pricing Authority to regulate the prices of 530 essential medicines.^31^ However, many Indian physicians prescribe branded generic drugs without concern for higher prices, or simply because they do not trust the quality of generics. Consequently, Indian patients devote 60% to 90% of their OOP healthcare spending to medicines whose costs have become a leading cause of impoverishment.^32^

## China

In 2009, China enacted an ambitious health reform aimed at increasing public health financing, providing essential drugs, expanding primary health facilities, and achieving UHC by 2020.^33^ The government’s recent investments in healthcare are part of a shift from export-led growth strategies to inclusive development strategies within the domestic market.^34^ Most new expenditures have subsidized urban and rural residents not already covered by the principal health insurance funds. In addition, in contrast to India, the government paid primary healthcare providers to deliver a minimum defined package of public health services and established the Essential Medicines Program.^35^

China’s domestic market is highly fragmented among over 5000 domestic manufacturers that export active pharmaceutical ingredients (APIs). Their production of final products, mostly generic medicines, is known for their uneven quality. Since the life sciences are a high priority for research and innovation, Chinese policy has promoted collaboration and technology transfer among domestic firms and Big Pharma by providing a favorable legal environment (through tax incentives), liberalization of drug prices and excellent conditions offered to highly qualified Chinese citizens returning from abroad.^36^ Also, since China’s healthcare reforms have increased healthcare expenditures, this has strengthened the attractiveness of the second largest domestic pharmaceutical market in the world. As of 2012, Big Pharma had already captured 20% of this market with each of the top 10 MNCs having invested between $3 billion and $9 billion.^37^

In contrast to Russia, 80% of medicines, in China, are distributed through public hospitals, which have until last year’s implementation of the zero mark-up policy, covered a large part of their budget through such sales.^38^ Chinese health policy today seeks to reduce the prices of all medicines, including those of Big Pharma, through a tendering process both at the central and provincial levels.^39^ Unfortunately, current tendering policies have created multiple areas of dysfunction ranging from high prices, low drug quality, irrational prescribing, access problems and discouragement of innovation due to slow and complicated drug approval procedures.^40,41^ Since 2011, as expenditures on pharmaceuticals have grown, price increases declined to 1% in 2016.^39^ To compensate for this downward pressure on prices, the government has agreed to accept new drugs from MNCs and to accelerate the approval process for innovative drug products, which in the past has often taken up to 7 years.^42^ Despite declines in profit margins of Big Pharma, prospects for continued profitability through higher sales volumes loom large, and as Ting Huang put it, the “dance between Big Pharma and the Chinese government will go on” [T. Huang unpublished data, 2017].

## Concluding Observations

From the perspective of Big Pharma, China represents the greatest opportunity to develop market share and draw on skilled human resources and great manufacturing potential. There is, of course, the risk of being subject to the central government’s insistence on assuring technology transfer in exchange for market access. Russia remains an important market, as the nation is likely to invest heavily in healthcare – at least in its wealthier regions. Beyond roughly half of the market now held by Big Pharma, Brazil will continue to provide fertile ground for mergers and acquisitions of national companies. Also, Brazil can serve as a testing ground for the expression of popular demand for newly branded pharmaceuticals. As for India, competition on quality in the branded generic and newly branded pharmaceuticals still represents an opportunity for Big Pharma as the government is open to MNCs and encourages investment, while remaining protective of its manufacturing base for generic medicines.

As we have noted, Big Pharma has developed different strategies to sell their medicines among the rapidly growing BRIC markets. Likewise, BRIC nations have responded in a manner that reflects their respective market size, institutions, negotiating power, and public health and industrial policies. These factors are critical in determining the potential role of BRIC nations as innovation hubs and the conditions affecting the implementation of their proclaimed policies. In the future, as in G7 nations, the role of government is likely to increase in attempting to improve access to state-of-the art medicines as well as the drug development process. We agree with Naci, Carter and Mossialos that “collective, concerted regulatory action is needed to send the correct signals to pharmaceutical companies.”^9^

The field of HPAM has focused mostly on hospitals, medical technologies and access to primary care. But as prescription drugs have become more effective and expensive, leaders in the field, worldwide, would do well to pay more attention to medicines and the drug development process. It is not sufficient to analyze these issues only from the point of view of pharmaceutical pricing strategies and reimbursement. Health policy analysts will need to reflect on the gaps among relevant theory, policy and practice^43^ and focus on pharmaceutical policy from a broader health system perspective.^44^ More research and attention to these issues will be useful in developing appropriate strategies to assure that healthcare systems obtain the prescription drugs they need on the most favorable terms possible.

## Acknowledgements

In conducting research for this paper, we benefited greatly from the contributions of Sudip Chowdhury and members of Wagner/NYU’s Seminar in Global Health Policy and Management: Katherine Baumann, Tinyan Huang, Anita Kishore, and Karen Matirosyan.

## Ethical issues

Not applicable.

## Competing interests

Authors declare that they have no competing interests.

## Authors’ contributions

VGR and GF provided overall framework and focused on Russia, India, and China. RFA focused on Brazil and on data for tables. All authors reviewed analysis and final text.

## Authors’ affiliations

^1^Robert F. Wagner Graduate School of Public Service, New York University, New York City, NY, USA. ^2^Université Paul Valéry Montpellier 3, IRIEC EA 740, Montpellier, France. ^3^Wagner School of Public Service, New York University, New York City, NY, USA.
